# Routine Metagenomics Service for ICU Patients with Respiratory Infection

**DOI:** 10.1164/rccm.202305-0901OC

**Published:** 2023-11-08

**Authors:** Themoula Charalampous, Adela Alcolea-Medina, Luke B. Snell, Christopher Alder, Mark Tan, Tom G. S. Williams, Noor Al-Yaakoubi, Gul Humayun, Christopher I. S. Meadows, Duncan L. A. Wyncoll, Richard Paul, Carolyn J. Hemsley, Dakshika Jeyaratnam, William Newsholme, Simon Goldenberg, Amita Patel, Fearghal Tucker, Gaia Nebbia, Mark Wilks, Meera Chand, Penelope R. Cliff, Rahul Batra, Justin O’Grady, Nicholas A. Barrett, Jonathan D. Edgeworth

**Affiliations:** ^1^ Centre for Clinical Infection and Diagnostics Research, Department of Infectious Diseases, School of Immunology and Microbial Sciences and; ^2^ Faculty of Life Sciences and Medicine, King’s College London, London, United Kingdom;; ^3^ Infection Sciences, Synnovis, London, United Kingdom;; ^4^ Department of Infectious Diseases and; ^5^ Critical Care Directorate, Guy’s and St Thomas’ NHS Foundation Trust, London, England;; ^6^ London School of Medicine and Dentistry, Queen Mary University, London, United Kingdom;; ^7^ UK Health Security Agency, London, United Kingdom; and; ^8^ Oxford Nanopore Technologies, Oxford, United Kingdom.

## Abstract

**Rationale:**

Respiratory metagenomics (RMg) needs evaluation in a pilot service setting to determine utility and inform implementation into routine clinical practice.

**Objectives:**

Feasibility, performance, and clinical impacts on antimicrobial prescribing and infection control were recorded during a pilot RMg service.

**Methods:**

RMg was performed on 128 samples from 87 patients with suspected lower respiratory tract infection (LRTI) on two general and one specialist respiratory ICUs at Guy’s and St Thomas’ NHS Foundation Trust, London.

**Measurements and Main Results:**

During the first 15 weeks, RMg provided same-day results for 110 samples (86%), with a median turnaround time of 6.7 hours (interquartile range = 6.1–7.5 h). RMg was 93% sensitive and 81% specific for clinically relevant pathogens compared with routine testing. Forty-eight percent of RMg results informed antimicrobial prescribing changes (22% escalation; 26% deescalation) with escalation based on speciation in 20 out of 24 cases and detection of acquired-resistance genes in 4 out of 24 cases. Fastidious or unexpected organisms were reported in 21 samples, including anaerobes (*n* = 12), *Mycobacterium tuberculosis*, *Tropheryma whipplei*, cytomegalovirus, and *Legionella pneumophila* ST1326, which was subsequently isolated from the bedside water outlet. Application to consecutive severe community-acquired LRTI cases identified *Staphylococcus aureus* (two with *SCCmec* and three with *luk* F/S virulence determinants), *Streptococcus pyogenes* (*emm1-*M1uk clone), *S. dysgalactiae* subspecies equisimilis (STG62647A), and *Aspergillus fumigatus* with multiple treatments and public health impacts.

**Conclusions:**

This pilot study illustrates the potential of RMg testing to provide benefits for antimicrobial treatment, infection control, and public health when provided in a real-world critical care setting. Multicenter studies are now required to inform future translation into routine service.

**At a Glance Commentary**

**Scientific Knowledge on the Subject**

Respiratory metagenomics (RMg) holds promise as a first-line diagnostic test for lower respiratory tract infections (LRTIs). In principle, it rapidly detects all potential pathogens along with antimicrobial resistance determinants and provides sequence typing for infection control or public health actions. Questions, however, remain on feasibility providing regular same-day testing and whether findings are sufficiently frequent and informative to justify translation into routine service.

**What This Study Adds to the Field**

We implemented a previously validated rapid RMg workflow into a daily pilot service for patients with community- and hospital-acquired LRTIs in general and specialist respiratory ICUs, performed alongside other routinely requested tests. RMg performance was comparable with findings from preceding research studies. Antibiotic treatment was optimized in almost half of the patients, but—perhaps more significantly—RMg revealed a hidden infectious burden in the ICU settings that was not reported by routine tests. Reasons included specific tests not being requested, fastidious culture requirements, or detection requiring sequencing information of either virulence factors or typing for more unusual and emerging infections. Although multicenter comparative studies are required, this study provides real-world evidence of how RMg could improve the initial management of LRTIs.

Community and hospital acquired lower respiratory tract infections (LRTIs) are caused by an expanding range of monomicrobial and polymicrobial infections. This presents significant challenges identifying or excluding microbial cause(s). Typically, samples are tested by different methodologies with results returned at different times over subsequent days (1). Culture is routinely used for bacterial identification, despite being slow, having suboptimal sensitivity particularly after antibiotic treatment, and its inability to detect many fastidious organisms. Multiplex PCR tests detect various pathogens and can add value to early decision-making, but they only target a restricted repertoire and cannot provide genomic detail or exclude infection.

Respiratory metagenomics (RMg) has the potential to become a first-line test for severe pneumonia, given its ability to identify essentially any microbe in a clinical sample along with antimicrobial resistance and virulence determinants (2). Retrospective and prospective proof-of-concept studies and case series have been published (3–9), but none have demonstrated feasibility and the breadth of impact from a single test as a routine daily service. We developed a 6-hour nanopore sequencing workflow (6) and determined performance characteristics and potential utility in a research setting for ventilated patients with coronavirus disease (COVID-19) (2). Here, we moved forward to determine the feasibility and clinical impact of a pilot RMg service, providing daily results to clinicians alongside routinely requested tests. Scientific and clinical oversight of this service improvement project was provided jointly by members of the diagnostic microbiology laboratory, the infectious diseases consult team, and the ICU.

Some of the results of these studies have been previously reported in preprint form (medRxiv, May 16 2023, www.medrxiv.org/content/10.1101/2023.05.15.23289731v1).

## Methods

### Setting and Sample Collection

RMg testing was offered to a 41-bed medical, surgical, and specialist respiratory ICU that included an extracorporeal membrane oxygenation service, Monday to Friday between November 22 and December 15, 2021, and between January 4 and March 25, 2022. Pilot service provision was agreed by the Critical Care Governance and Audit Committee under the NHS Quality Improvement and Patient Safety (QIPS) governance process as previously described (10) (QIPS Reference 2021:13023). Duty intensivists selected mechanically ventilated (MV) patients to have additional RMg testing alongside culture and 16S ribosomal RNA (rRNA) sequencing that was performed at a referral clinical laboratory, with results returned within 4–6 days after acute treatment decisions had been made. Sample selection criteria were based on the potential for a rapid result to assist with diagnosis or exclusion of LRTIs and antibiotic prescribing. Respiratory samples were retrieved from the ICU at 8:30 a.m., with the goal of receiving results before 5 p.m. Representative severe community-acquired LRTI (CA-LRTI) cases are also presented from the first 8 weeks of the following influenza season (Winter 2022), given that CA-LRTIs were rare during the pilot service conducted during the COVID-19 pandemic.

### RMg Sequencing Workflow

**Figure 1.**
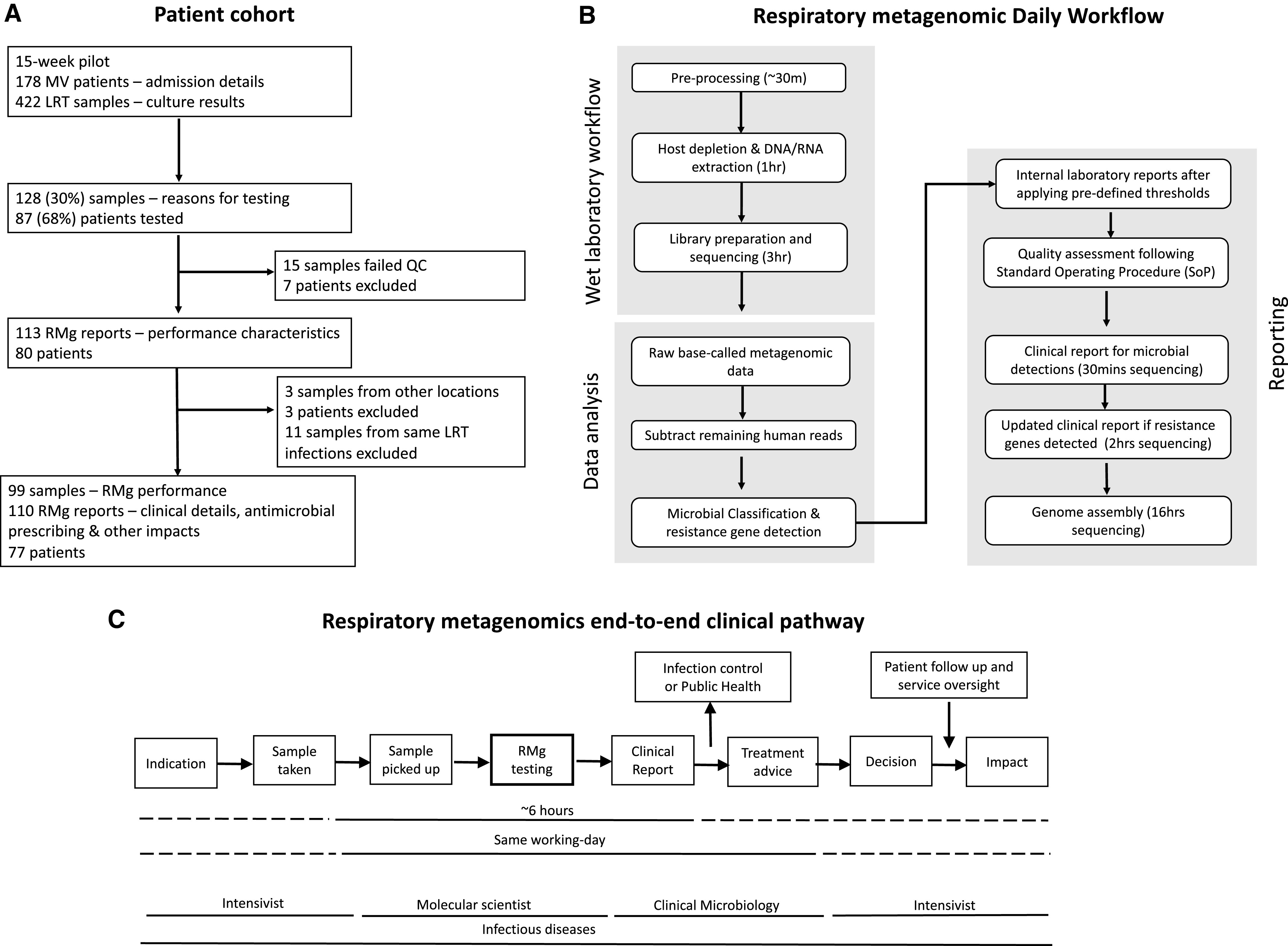
Schematic overview of the study. (*A*) Overview of the patient cohort and sample set included in the respiratory metagenomics (RMg) pilot service. (*B*) The metagenomics regimen that was followed on a daily basis when samples were requested for RMg service. Steps outlined include sample collection until reporting results to ICU physicians. (*C*) Respiratory metagenomic end-to-end clinical pathway. LRT = lower respiratory tract; MV = mechanically ventilated; QC = quality control.

In total, 128 samples were processed for RMg sequencing, which included 111 BALs, three tracheal aspirates, eight nondirect BALs (NDLs), and six pleural fluids. RMg testing involved saponin-based host depletion, microbial extraction, library preparation, and nanopore sequencing as previously described (2, 6, 11) (Figure 1). Every RMg run also included quality controls (no template control [NTC], positive control [PC] and a competitive spiked-in internal control [IC]) to identify run or single-sample failures and contamination. The method is detailed elsewhere (*see* the online supplement).

Time-point data analysis was performed using an in-house pipeline (https://github.com/GSTT-CIDR/RespiratoryCmg) incorporating taxonomic classification, antimicrobial resistance (AMR) gene detection and sequence-based typing (SBT). The *k*-mer–based classifier Centrifuge (12) was used for microbial identification with the U.S. Food and Drug Administration Database for Reference *G*rade Microbial Sequences (or, FDA-ARGOS) database (13), curated with only respiratory organisms containing 673 microbial sequences.

#### Reporting of RMg results

Sequencing reports generated at 30 minutes and 2 hours were interpreted following the standard operating procedure. Bacteria were reported using 30 minutes of sequence data when representing ⩾1% total microbial reads and fungi if five or more reads were detected and with a Centrifuge score of 8,000 or higher. The Centrifuge score is defined as the aggregated score of the number *k*-mers from a sequencing read matching perfectly an organism’s reference sequence in the database (12). Microorganisms reported from the RMg workflow in this study were referred to as “respiratory pathogens” or “pathogens,” and they were defined as microorganisms causing respiratory infection in ICU patients. A list of reportable organisms was compiled and followed for reporting. The list was based on previous LRTI studies (1, 2, 12–14) and previous findings from the archives of microbiological culture in the past 5 years collected from the clinical laboratory (*see* Table E1 in the online supplement). Organism-specific reporting criteria were also set for certain organisms (such as *E. faecium* and anaerobes). The reporting is described in detail in the online supplement.

Reports on acquired-resistance genes restricted to extended-spectrum β lactamases in *Enterobacterales*, SCCmec in *Staphylococcus aureus*, and vancomycin-resistance gene clusters in *Enterococcus faecium* were included in the 2-hour sequence reports (2).

Clinical sequence reports were uploaded in PDF format to the ICU electronic health record after scientific and medical review. Results were also communicated by e-mail or verbally to the duty intensivist and infectious diseases doctor (Figure 1C). RMg performance was compared with routinely requested tests. Discrepant results were investigated using routine 16S rRNA sequencing testing or an in-house pathogen-specific targeted quantitative PCR (qPCR).

### Data Availability

Sequencing data presented in this study are available in the European Nucleotide Archive under project number PRJEB59568.

### Patient Management and Impacts of RMg

Clinical microbiology and antimicrobial prescribing data were collected prospectively from all MV ICU patients who had at least one LRT sample collected during the pilot study alongside RMg. Samples from patients in nonpilot critical care areas were excluded from downstream analysis and data collection. RMg-based antimicrobial treatment changes and findings of infection control or public health importance were communicated by e-mail or phone calls the same day. RMg results and impacts were reviewed biweekly by a multidisciplinary team of three intensivists, an infectious disease doctor, two microbiologists, and a pharmacist. Clinical implications of unexpected or discrepant results or any adverse impacts of interventions in response to RMg results were reviewed.

## Results

### Clinical and Microbiological Characteristics of Ventilated ICU Patients

A total of 172 ventilated patients admitted to the ICU during the 15-week period had 422 LRT samples cultured. In total, 128 of 422 (30%) LRT samples from 87 of 172 (51%) patients had additional RMg testing (Figure 1A). The clinical characteristics of RMg-tested patients were similar to those of nontested patients, apart from more COVID-19 infections (33% vs. 21%) and extracorporeal membrane oxygenation therapy (38% vs. 5%). (Table 1). Nine of 172 (5%) patients had any LRT sample growing Gram-negative bacteria phenotypically resistant to first-line empiric treatment of hospital-acquired LRTI (piperacillin–tazobactam), and 3 patients had vancomycin-resistant *E. faecium* (VRE) or carbapenem-resistant *P. aeruginosa.* No cases of methicillin-resistant *S. aureus* or carbapenem-resistant Enterobacterales were reported (*see* Table E2 in the online supplement).

**Table 1.** Clinical Characteristics and Routine Microbiological Testing of the Patient Cohort During the 2021–2022 Winter Season

Characteristic	Patients with RMg (*n* = 87)*	Patients without RMg (*n* = 85)
Patient details		
Age, median (IQR)	53 (39–65)	62 (53–71)
Sex, *n* (%)	30 (34)	27 (32)
ECMO, *n* (%)	33 (38)	4 (5)
Reason for admission, *n* (%)		
Respiratory infection		
COVID-19	29 (33)	18 (21)
LRTI (other)	20 (23)	17 (20)
CAP	7 (8)	4 (5)
Medical (nonrespiratory)	13 (15)	14 (17)
Cardiothoracic surgery	7 (8)	16 (19)
Other surgery	7 (8)	11 (13)
Septic shock	4 (5)	5 (6)
Respiratory culture^†^		
No. of LRT samples	242	180
Gram-negative organisms	99 (41)	76 (42)
Gram-positive organisms	35 (14)	31 (17)
* Candida* spp.	85 (35)	67 (37)
* Candida* spp. only	50 (21)	36 (20)
* Aspergillus* spp.	2 (1)	0 (0)
No growth or URTF	81 (33)	48 (27)

*Definition of abbreviations*: CAP = community-acquired pneumoniae; ECMO = extracorporeal membrane oxygenation; IQR = interquartile ranges; LRT = lower respiratory tract; URTF = upper respiratory tract flora.

* Clinical details at the time of each RMg test presented in Data File E2. These include patients excluded postdownstream data analysis.

^†^ Organisms cultured from all LRT samples collected during the 15-wk study period are listed in Table E2.

### RMg Performance against Routine Testing

RMg was performed on 128 samples that were sent for new suspected community-acquired (CA) LRTIs (23%), at the start of (29%) or during (34%) an episode of suspected hospital-acquired (HA) LRTI or for other reasons (14%) (Table 2). Fifteen of 128 samples from 7 patients failed quality control (*see* Table E3 in the online supplement) and so were excluded from further analysis, along with three samples from 3 patients taken while on non-ICU acute wards. All remaining 110 samples from 77 patients (96 BALs, three tracheal aspirates, six NDLs, and five pleural fluids) were included in clinical impact evaluations, but 11 of the 110 samples (three NBLs and eight BALs) that were repeat LRT samples during the same infection episode were excluded from RMg performance calculations. Therefore, RMg performance was calculated on the remaining 99 LRT samples (Figure 1A; Data Files E1 and E2).

**Table 2.** Antimicrobial Treatment Changes in Response to RMg Results

Treatment Change	*n* (%)
Indication for RMg testing	*N* = 128
CA LRTI	30 (23)
HA LRTI	80 (63)
Start of episode	37 (29)
During episode	43 (34)
Other*	18 (14)
Antibiotic prescribing^†^	*n* = 110^‡^
Receiving antibiotics start of RMg test day	89 (81)
Receiving antibiotics end of RMg test day	98 (89)
Deescalation, antibiotics stopped with:	
RMg^§^	29
Same day	7 (24)
Next day^ǁ^	22 (76)
Meropenem	15
Piperacillin–tazobactam	6
Linezolid	5
Other	3
Escalation, antibiotics started with:	
RMg^¶^	*n* = 24
Same day	21 (87)
Next day**	3 (13)
Meropenem	10
Linezolid	4
Other	10

*Definition of abbreviations*: CA = community-acquired; HA = hospital-acquired; LRTI = lower respiratory tract infection; RMg = respiratory metagenomics.

* Includes where the focus of infection was uncertain at time of testing, to exclude respiratory infection preimmunomodulation or after completing therapy for LRTI.

^†^ Fifteen patients had antibiotics started or stopped for reasons not linked with the RMg result.

^‡^ Excludes samples failed at quality control (*n* = 15) or sent from nonpilot critical care areas (*n* = 3).

^§^ Antifungals were stopped in 3 patients in response to RMg results.

^ǁ^ Two patients had antibiotics stopped on second day after testing.

^¶^ Antifungals were started for 5 patients in response to RMg results.

** Three patients had antibiotics started in response to RMg results 2 days after receipt of the result.

The median turnaround time from sample receipt to RMg reporting was 6.7 hours (interquartile range = 6.1–7.5 h; maximum, 30.5 h), with 90% having same-day final reports. This compared with verbal communication of the interim culture results when available on the following afternoon (median, 29 h) and final reports generated at a median of 40 hours.

In 39 of 42 culture-positive samples, RMg was in agreement with culture findings. RMg missed culture-reported organisms in three of 42 samples. All missed organisms were reported as scanty growth by culture (*S. aureus* [P2 and P77] and *K. pneumoniae* [P7]) (*see* Figure E1 in the online supplement). RMg did not detect clinically relevant organisms in 46 of 57 samples reported as “negative for pathogens” by culture. These included 23 of 46 samples reported as “negative” or “no growth” by culture and 23 of 46 samples reported positive for commensal or non–clinically relevant organisms, such as *Candida* spp. (Figure E1). Clinically relevant organisms were detected by RMg in 11 of 57 samples; these included *S. aureus* (*n* = 3), *E. faecium* (*n *= 2), anaerobic bacteria (*n* = 4), *C. striatum* (*n* = 1), and *C. koseri* (*n* = 1).

On the basis of these findings, RMg was 93% (95% confidence interval [CI], 81–99%) sensitive and 81% (95% CI, 68–90%) specific on a per-sample basis compared with culture, as per the guidelines of the UK Standards for Microbiology Investigations (14). Only three of the 11 findings were not confirmed by confirmatory testing (*see* Table E4 in the online supplement), which increased the specificity of RMg to 94% (95% CI, 83–99%). Overall, 26 of 99 (26%) samples contained organisms that were only reported by RMg, of which 15 were otherwise culture positive and 11 were culture negative. These included DNA viruses (herpes simplex virus 1 [HSV-1], *n* = 4; and cytomegalovirus [CMV], *n* = 1), bacteria (*n* = 8), and bacteria plus HSV-1 (*n* = 1) (Data File E1). Considering additional findings that were reported by RMg only as “false-positive detections,” specificity was 74% (95% CI, 64–82%). Additionally, on a per-sample–type basis, RMg was 92% sensitive (95% CI, 79–98%) and 81% specific (95% CI, 68–91%) for BALs and NDLs only (*n* = 92/99).

RMg findings were also compared with routine referral 16S rRNA sequencing. In total, 75 of 99 RMg samples also had 16S rRNA sequencing, of which 46 (61%) were concordant. These included 29 of 46 true-negative RMg samples for which neither test reported any pathogens. Of the remaining samples, 21 of 75 (28%) were discordant, and for eight of 75 (11%) samples, both tests, were in agreement for ⩾1 microbial detection (Data File E2).

**Figure 2.**
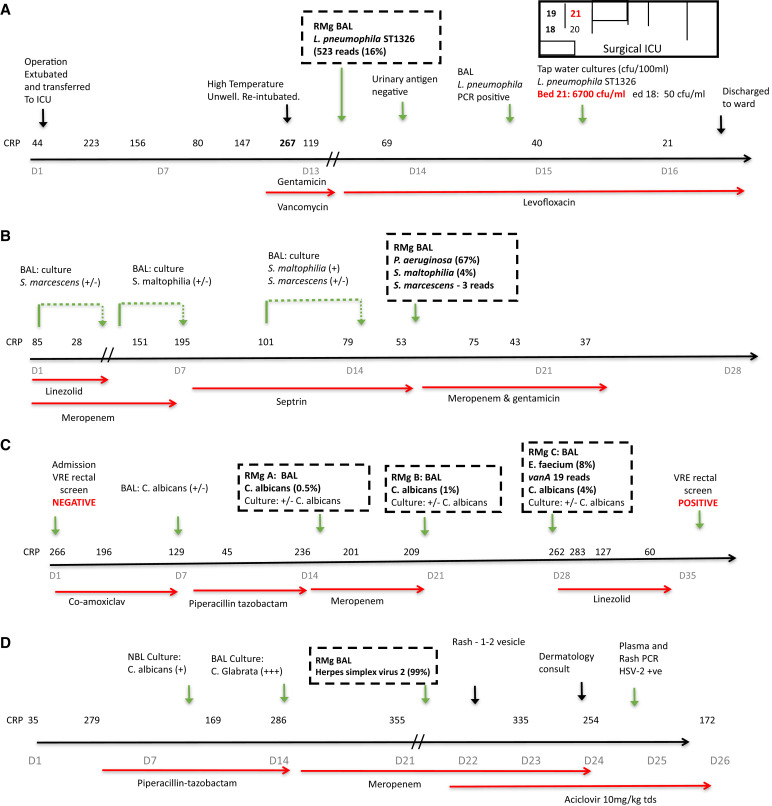

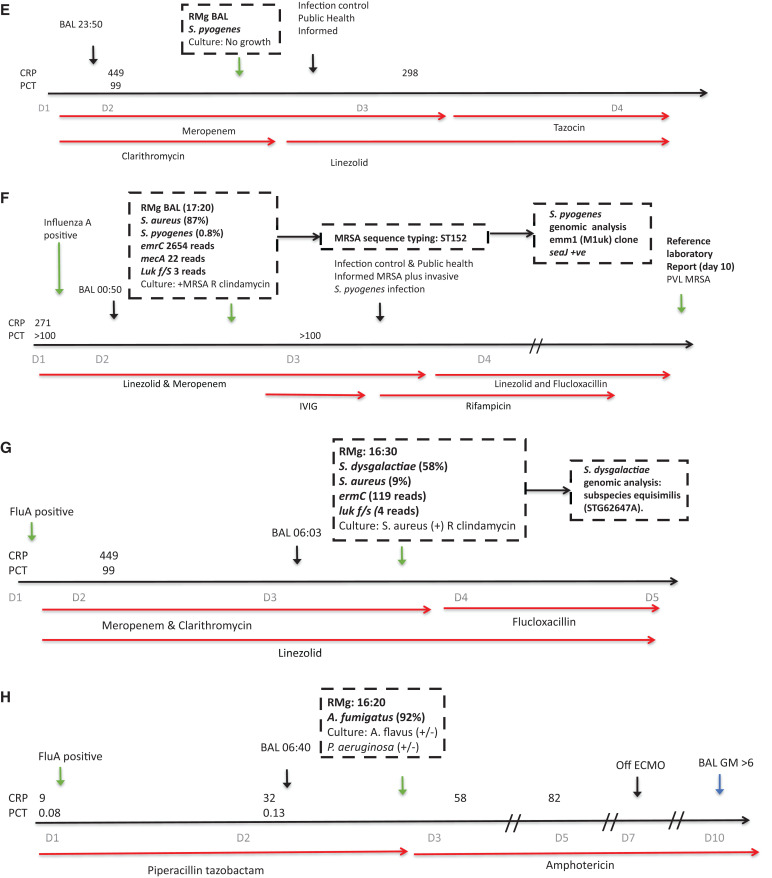
Patient ICU timelines illustrating the integration of RMg results into antimicrobial treatment and infection control decisions. Hospital-acquired lower respiratory tract infection (LRTI): (*A*) ICU-acquired *L. pneumophila* ST1326 pneumonia. Unexpected bacteria prompting antibiotic escalation, infection control, and public health interventions. (*B*) *P. aeruginosa* ventilator-associated pneumonia (VAP)–LRTI. New bacterial pathogen in patient with severe COVID-19 pneumonitis prompting antibiotic escalation. (*C*) ICU-acquired vancomycin-resistant *E. faecium*. Unexpected antimicrobial resistance (AMR) bacteria with patient and infection control impact. (*D*) Unexpected disseminated reactivation of herpes simplex virus 2 (HSV-2). Community-acquired LRTI: (*E*) Influenza with secondary *S. pyogenes* infection. (*F*) Influenza with secondary Panton–Valentine leukocidin and methicillin-resistant *S. aureus* (PVL-MRSA) and *S. pyogenes* infection. (*G*) Influenza with secondary PVL and methicillin-sensitive *S. aureus* (PVL-MSSA) and *S. dysgalactiae* infection. (*H*) Influenza with secondary invasive aspergillosis prompting urgent treatment. The details of each case are presented in the online supplement. CRP = C-reactive protein; CFU = colony-forming unit; D = day.

Acquired-resistance genes were also reported in five samples, *vanA* (P88), bla*_SHV_* (P96, P115, and P122), and bla*_CXT-M_* (P117). RMg missed *K. pneumoniae* bla*_CXT-M_* in one patient (Figure 2D and sample P110), but it was identified by repeat sequencing using MinION Flow Cell, which has higher sequencing yields (15) (Data File E1).

### Impacts on Antibiotic Treatment

RMg contributed to prescribing decisions in 88 of 110 (80%) cases (Table 2). In 24 (22%) cases, antibiotics were started (*n* = 10) or escalated (*n* = 14) on the basis of detecting organisms with intrinsic (*n* = 20) or acquired (*n* = 4) resistance to current therapy, the majority (87%) that day (Table 2). In 29 (26%) cases, antibiotics were deescalated or stopped predominantly on the next morning ward round (*n* = 22/29 cases; 76%). Deescalation occurred in 66% (19/29) of cases when RMg detected no clinically relevant organisms (13 detected no organisms, three detected upper respiratory tract commensals, and three detected *Candida spp.*). All deescalation cases were followed up. One patient (P78) had antibiotics restarted to treat *P. aeruginosa* cultured from a respiratory sample taken 3 days after RMg informed the stopping of antibiotics. The patient was otherwise progressing well and left the ICU a few days later. Clinical details of all cases are given in Data File E2, with representative timelines for three HA LRTI cases in Figures 2A–2C.

For 35 (32%) patients, antimicrobials were not changed, but contributions to prescribing decisions were recorded, mostly by reassuring clinicians of no unexpected pathogens. Thereby preventing escalation on antimicrobial treatment, particularly in heavily immunosuppressed patients and in patients with persistent inflammation on antimicrobial treatment (*n* = 11). RMg results also prompted early immunomodulation for suspected inflammatory lung conditions (*n* = 7) after excluding pathogens (Table 3).

**Table 3.** Categorizing Impact of Respiratory Metagenomic Results on Antibiotic Prescribing

Treatment Category	Organisms Identified by RMg*	Antibiotic Changes (*n*)
Start or escalate to active antimicrobials (*n* = 24, 22%)	AmpC Enterobacterales (9), anaerobes (3), *Candida* spp. (2), *P. aeruginosa* (3), VRE (1), ESBL *K. pneumoniae* (1), *H. influenza* and *K. variicola* (1),^†^ *H. influenza* and *M. morganii* (1), *E. faecium* (2), and *C. striatum* (1)	Started: meropenem (10), linezolid (3), linezolid and anidulafungin (1), ciprofloxacin (1), piperacillin–tazobactam (2), ciprofloxacin (1), and other (6)
De-escalate or stop (*n* = 29, 26%)	No significant organisms (29), comprising no organisms (13), *Candida* spp. (3), URTF (3), anaerobes (3), MSSA (2), *K. variicola* and *H. influenzae* (3), *E. faecalis* (1), and *E. coli* (1)	Stopped: meropenem (6), piperacillin–tazobactam (4), piperacillin–tazobactam and gentamicin (2), linezolid (1), meropenem and linezolid (4), meropenem and other (5), co-amoxiclav (1), ciprofloxacin (1), levofloxacin (2), and other (3)
Prevent escalation, reassure, or inform other therapy (*n* = 35, 32%)	No significant organisms, comprising no organisms (10), *Candida* spp. only (3), URTF (2), anaerobes (6), *K. pneumoniae* (3), other Enterobacterales (4), MSSA (4), *Candida* spp. (with MSSA) (1), *C. striatum* (1), and *E. faecium* (1)	Reassure correct antimicrobial chosen (17), exclude organisms to bring forward immunomodulation (7), exclude untreated organisms in complex or immunosuppressed patients (8), and persistent inflammation on current antibiotics (3)
No treatment impact (*n* = 22, 20%)	No significant organisms: No organisms (11); *C. albicans* (2); *K. pneumoniae* (3); other Enterobacterales (3); and 1 each of *P. aeruginosa*, *E. faecalis*, and MSSA (3)	Clinical concern prevented deescalation in response to RMg result (6); potential or proven infection at other site (8); delay returning result (4); missed information (resistance) (2); quantity of organism (1); and organism missed (1)

*Definition of abbreviations*: ESBL = extended spectrum β-lactamases; MSSA = methicillin-sensitive *S. aureus*; RMg = respiratory metagenomics; URTF = upper respiratory tract flora; VRE = vancomycin-resistant *E. faecium*.

Impact categories were as follows: *1*) earlier appropriate antimicrobials where the result prompted changes to existing therapy to target the identified organism(s); *2*) deescalating antimicrobials where the result contributed to stopping or narrowing antimicrobial spectrum; *3*) prevent antimicrobial escalation, reassuring that current therapy was appropriate, or informing nonantimicrobial treatment therapy; and *4*) no identified benefit for range of reasons.

* Organisms identified by RMg in monomicrobial and polymicrobial LRT samples.

^†^ *K. variicola* and *H. influenza* were grown, but susceptibilities were not available. *H. influenza* was resistant to coamoxiclav, but the patient showed improvement and was extubated so the patient completed the 5-day course. Coamoxiclav was started 2 days later.

Anaerobes detected in 12 samples (10 BALs and two pleural fluids) were deemed clinically relevant on the basis of clinical findings and the absence of alternative plausible pathogens. Patients with CA LRTI (*n* = 8) had a history of aspiration, and patients with HA LRTI (*n* = 4) had received antimicrobials for 5–12 days, some of which lacked anaerobic activity. Antibiotics were started (*n* = 4), deescalated (*n* = 3), or continued (*n* = 5). Conversely, their exclusion from a clinically suspected lung abscess and empyema prompted a diagnosis of lung infarction with hydropneumothorax and shortening of the planned antibiotic course from 6 weeks to 5 days (*see* Table E5 in the online supplement).

Twenty-two of 110 (20%) RMg findings had no recordable impact, either because infection was diagnosed at another site (*n* = 8), results were not acted on (*n* = 6), or decisions were made before results were returned (*n* = 4) (Table 3).

### Information for Infection Control

Same-day communication of one case of VRE and two cases of extended-spectrum β-lactamases prompted the early institution of barrier precautions. The VRE (P88; Figure 2C) was only cultured from a rectal swab requested during follow up, in response to RMg results. Additionally, *K. variicola* (3 patients) transmission on the basis of overlapping ward stays was investigated using RMg data. *K. variicola* from 2 of 3 patients had 18% of the genome shared with 99.9999% genetic similarity patient-to-patient transmission (genetic similarity was also confirmed by whole-genome sequencing of the isolate where 90% of the genome was shared between samples).

### Unexpected Organisms Reported by RMg

RMg reported organisms that were not generally detectable by tests requested during the initial patient investigation in nine samples that were classified as unexpected. *L. pneumophila* ST1326 (serogroup 10) was an ICU-acquired infection 13 days postcardiothoracic surgery (P123), which was confirmed by PCR but not urinary antigen testing. The same sequence type was isolated from the adjacent hand-basin tap water (Figure 2, patient A), so new water filters were fitted to prevent further cases. *M. tuberculosis* that was detected in a patient admitted with hemoptysis a few months after starting antituberculous therapy was interpreted as dead organisms, so no action was taken. That sample and four further samples were auramine and culture negative. *Tropheryma whipplei* (P103) was detected in a patient with HA LRTI after a thymoma resection, prompting ceftriaxone treatment and follow-up by an infectious diseases doctor, but the significance remained uncertain. CMV detection in a patient with Jo-1 antisynthetase deficiency prompted plasma viral load testing, the results of which were positive (log 3.5–3.6). It was considered clinically relevant, and treatment with ganciclovir was commenced (P35). HSV-1 was detected in five samples, but all were considered nonpathogenic reactivation.

### Representative Cases from the 2021–2022 Winter Season

Eight severe CA LRTI cases were admitted over an 8-week period, of which six cases were coinfections with influenza (*see* Table E6A in the online supplement). RMg identified Panton–Valentine leukocidin–*S. aureus* (three cases), *S. pyogenes* (two cases), *S. pneumoniae* (two cases), *S. dysgalactiae* (one case), *L. pneumophila* (one case), and *A. fumigatus* (one case). Only one *Streptococcus* species (*S. pneumoniae*) was cultured. Treatment was escalated in three cases that day (the addition of linezolid, intravenous immunoglobulin, or ambisome (Figures 2E–2H). Panton–Valentine leukocidin–*S. aureus* and *S. pyogenes* cases were reported to public health officials that day. Subsequent analysis of RMg data identified one case of *S. pyogenes* as an *emm1-*M1uk clone and the *S. dysgalactiae* as subspecies *equisimilis* (STG62647A).

Sixteen additional patients had RMg testing, the most important result of which was unexpected HSV-2 in a patient with new hepatitis and suspected drug rash postvascular surgery (Figure 2, patient D; Table E6). High-dose acyclovir was started that day. Subsequent plasma and rash swab samples were HSV-2 positive.

## Discussion

There are frequent calls to expedite the evaluation of metagenomic testing for acutely unwell patients (16). We need to determine how early comprehensive pathogen information can be made clinically valuable by improving antimicrobial prescribing and other infection control or public health interventions, rather than the dominant outcome being frequent treatment of clinically irrelevant components of the microbiome that promotes further antimicrobial resistance. We, therefore, evaluated RMg here after detailed assessment of performance characteristics, incorporation of quality controls, and positivity thresholds chosen to avoid major errors (2, 6). We also provided RMg prospectively alongside the routine microbiology service, interpreted by the ICU infectious diseases consult team and the duty intensivist, so results were incorporated into daily clinical decision-making. The microbiology laboratory, infectious diseases department, antimicrobial pharmacy, infection control, and ICU were all part of the new-service evaluation team to ensure seamless communication of all relevant information between the laboratory and clinical teams. This was combined with oversight from a biweekly multidisciplinary review group that monitored prescribing and any unexpected safety signals. Thus, given the complex behavioral framework around interpreting novel molecular tests, balancing concerns to ensure treatment with appropriate antimicrobials without adverse societal impact of driving AMR (17), we concluded that it was now both appropriate and necessary to begin evaluating metagenomics in a structured real-world setting.

There were five overlapping categories impacted by RMg. The first category was the earlier provision of RMg results than what culture usually provides (median: 6.7 h from RMg vs. 40 h from culture), thus improving initial antimicrobial treatment. This occurred in almost half of the patients and was predominantly due to species identification rather than acquired-resistance genes, which were uncommon in this cohort. Second was the identification of organisms that are hard to identify by culture or are suppressed by prior antibiotics. Anaerobes with or without *S. milleri* were found in 10% of samples, findings similar to those of a previous study using the same service framework (10), and they have been identified in other RMg studies (7). Anaerobes have been considered respiratory pathogens (7, 18–20), although their significance has more recently been questioned (21), given the recognition that they are part of a healthy respiratory microbiome (22, 23). Their absence from microbiology reports because of fastidious culture requirements has prevented detailed clinic-pathological correlation, so this metagenomic approach can prompt reassessment of their significance when identified above reporting thresholds. In cases where they are considered the causative agent, there would be an opportunity for useful antibiotic deescalation rather than continuing on broad-spectrum antibiotics without an identified pathogen. The third category is the reporting of no (significant) organisms to provide an actionable “negative” result in appropriate clinical contexts, which RMg is uniquely placed to provide. No adverse consequences were identified when deescalation took place in response to “negative” results. However safe deescalation always requires close monitoring, confidence in sample quality, and an understanding of methods limitations and reporting thresholds. This is particularly relevant in acutely unwell patients or when considering immunomodulation for clinically suspected inflammatory lung conditions. The fourth category is the identification of antimicrobial-resistant (AMR) organisms for infection control. One VRE and two extended-spectrum β-lactamase cases prompted the early institution of barrier precautions. Detection of the VRE was unlikely without RMg testing. Furthermore, clinical adjudication of discordant RMg cases during biweekly review meetings concluded that no adverse consequences were caused. In particular, RMg false-negative cases (missed bacteria being reported as scanty growth in polymicrobial samples) did not cause adverse outcomes. Also, adjudication concluded that the significance of not reporting these organisms was unclear.

These first four categories represent potential improvements to the routine culture pathway, but the final category was the identification of organisms currently requiring targeted molecular tests that were not requested by intensivists. Some proved to be clinically important such as the CMV, HSV-2 and *L. pneumophila* cases, with the latter prompting interventions to prevent further cases. In contrast, the five HSV-1, *M. tuberculosis*, and probably the *T. whipplei* cases were not significant, although all would be in different clinical contexts (24–27). Providing unexpected or unrequested results can prompt unnecessary investigation and treatment; however, identifying benefits in about a third of cases as found here could be considered an acceptable yield.

The utility of this fifth “molecular” category is extended when the additional information provided by pathogen sequencing is considered. Targeting the water supply as the source of *L. pneumophila*-ST1326 required sequence-based typing, not just organism identification, as did confirming *K. variicola* transmission (2). RMg applied to severe CA LRTI cases identified virulence factors (*luk f/S*) and emerging virulent clones missed by culture (*S. dysgalactiae* STG62647A and *S. pyogenes emm1-*M1uk) (28). The latter is of particular public health significance, given its link with severe pediatric infections and deaths, first announced by UK Health Security Agency 8 weeks after this case (29, 30). Finally, demonstrating the ability to assess genotypic azole resistance in the *A. fumigatus* sequence from RMg data would be a significant improvement to current fungal AMR testing.

This study has limitations. Only three samples maximum were processed per day because of limited operator availability, so the proportionate impact may be reduced when extended to all respiratory samples. The reported sample-failure rate (12%) was mostly due to a defective DNA-extraction batch or operator-introduced contamination, which is a recognized current limitation of RMg (31, 32). This can be addressed by reducing hands-on time by using automation. Only phenotypes from acquired-resistance elements were reported; however, expansion to phenotypic prediction caused by other AMR mechanisms (mutational) to increase the usability of RMg in settings with higher AMR rates is required. This workflow is not designed to detect RNA viruses. Therefore assessing a modified version of this workflow that detects RNA viruses, or assessing other workflows that additionally detect RNA viruses, is necessary to extract the full value from RMg (33, 34). Finally, because of the lack of a noninfectious control group, the pathogenicity of certain organisms could not formally assessed, beyond considering all available clinical information at the bedside during their stay on ICU.

In conclusion, this pilot service demonstrates the clinical utility of RMg testing in a routine setting, reporting organisms that are usually detected by culture alongside fastidious and/or uncultivable organisms while also providing genomic information for AMR prediction and/or identifying hospital-acquired infections and emerging hypervirulent community clones aiding decision-making with regard to infection control. Realizing all these benefits for individual patients and the wider healthcare system will require change to current practice by many professional groups working more closely together in the same acute timeframe (35). Clinical implementation still requires further technology refinement, addressing accreditation and regulatory requirements, along with gathering data from larger multicenter studies and health and economic analyses. These studies should include comparisons with usual practice and the application of RMg to infectious and noninfectious patients to better inform the distinction between colonization and infection and quantify clinical impacts (32, 36). Nevertheless, given recognized gaps in preparedness highlighted by the COVID-19 pandemic (37–39) and increasing AMR (40, 41), this study gives both encouragement and urgency to introduce metagenomics for evaluation as the standard of care in acute care pathways (16, 42).
